# Supplementary material for: Nf1 loss promotes Kras‐driven lung adenocarcinoma and results in Psat1‐mediated glutamate dependence
Source: EMBO Mol Med. 2019 Apr 29;11(6):e9856. doi: 10.15252/emmm.201809856 (PMC6554671; doi:10.15252/emmm.201809856)

# APPENDIX FOR
# *Nf1* Loss Promotes *Kras*-Driven Lung Adenocarcinoma and Results in Psat1-Mediated Glutamate Dependence

## Contents

APPENDIX TABLES ....................................................................................................2

Appendix Table S1. Single-Guide RNA Sequences ....................................................................................................2

Appendix Table S2. Primer Sequences in qRT-PCR Analyses....................................................................................................2

Appendix Table S3. P-Values ....................................................................................................3

APPENDIX FIGURE LEGENDS....................................................................................................9

Appendix Figure S1. Nf1-Silenced Xenograft Tumors Display Selective Growth Advantage ....................................................................................................9

Appendix Figure S2. Full Experimental Data Supporting Figure 2 ....................................................................................................9

Appendix Figure S3. Nf1 Silencing Induces Fak1 Activation .................................................................................................... 10

Appendix Figure S4. Nf1-Silenced Cells are More Glycolytic.................................................................................................... 11

Appendix Figure S5. Glutaminase and Aminotransferase Inhibition Specifically Inhibits Nf1-Mutant Cell Proliferation.................................................................................................... 11

Appendix Figure S6. Fak1 Activity Required for Sensitivity to Glutaminase and Aminotransferase Inhibition 12

Appendix Figure S7. Full Experimental Data Supporting Figure 6 .................................................................................................... 13

APPENDIX FIGURES.................................................................................................... 15

Appendix Figure S1.................................................................................................... 15

Appendix Figure S2.................................................................................................... 16

Appendix Figure S3.................................................................................................... 17

Appendix Figure S4.................................................................................................... 18

Appendix Figure S5.................................................................................................... 19

Appendix Figure S6.................................................................................................... 20

Appendix Figure S7.................................................................................................... 21

# APPENDIX TABLES

**Appendix Table S1. Single-Guide RNA Sequences**

| **sgRNA** | **Sequence** |
|---|---|
| *sgTom* | 5'-GGCCACGAGTTCGAGATCGAGGG-3' |
| *sgNf1.1* | 5'-GAACTGGGTAGAAAATTATCC-3' |
| *sgNf1.2* | 5'-GATTATCCGAATTCTTAGCA-3' |
| *sgNf1.3* | 5'-GTCCGAAGTTCGGCTGCATGT-3' |
| *sgFak1.1* | 5'-CCAGTCTACAGATTTGATA-3' |
| *sgPsat1* | 5'-CTGTCCTCGAACCAGTCGAT-3' |

**Appendix Table S2. Primer Sequences in qRT-PCR Analyses**

| **Gene** | **Forward primer** | **Reverse primer** |
|---|---|---|
| *Psat1* | 5'-CAGCCTGTCAGCCATCTTTG-3' | 5'-CGTGAGTCGCTGTTGACTTG-3' |
| *Areg* | 5'-TCTCCACAGGGGACTACGAC-3' | 5'-AGCCGGATATTTGTGGTTCA-3' |
| *Peg10* | 5'-GGACCCCTCATCCTTCGT-3' | 5'-TTCTTAAAACCCGCCTGTTC-3' |
| *Gapdh* | 5'-GTGGCAAAGTGGAGATTGTTGCC-3' | 5'-GATGATGACCCGTTTGGCTCC-3' |

**Appendix Table S3. *P*-Values**

| Figure panel | Comparison and statistical test | *P*-value |
|---|---|---|
| Figure 1 (B) | 20 weeks post-infection: sgTom vs. sgNf1.1 *t*-test | $P < 0.0001$ |
| | 20 weeks post-infection: sgTom vs. sgNf1.2 *t*-test | $P < 0.0001$ |
| | 20 weeks post-infection: sgTom vs. sgNf1.3 *t*-test | $P < 0.0001$ |
| Figure 1 (C) | 20 weeks post-infection: sgTom vs. sgNf1.1 *t*-test | $P < 0.0001$ |
| | 20 weeks post-infection: sgTom vs. sgNf1.2 *t*-test | $P < 0.0001$ |
| | 20 weeks post-infection: sgTom vs. sgNf1.3 *t*-test | $P < 0.0001$ |
| Figure 1 (D) | 21 weeks post-infection: sgTom vs. sgNf1.1 *t*-test | $P < 0.0001$ |
| | 21 weeks post-infection: sgTom vs. sgNf1.2 *t*-test | $P < 0.0001$ |
| | 21 weeks post-infection: sgTom vs. sgNf1.3 *t*-test | $P < 0.0001$ |
| Figure 1 (F) | 21 weeks post-infection: sgTom vs. sgNf1.1 *t*-test | $P < 0.0001$ |
| | 21 weeks post-infection: sgTom vs. sgNf1.2 *t*-test | $P = 0.0003$ |
| | 21 weeks post-infection: sgTom vs. sgNf1.3 *t*-test | $P = 0.0001$ |
| Figure 1 (G) | 21 weeks post-infection: sgTom vs. sgNf1.1 *t*-test | $P < 0.0001$ |
| | 21 weeks post-infection: sgTom vs. sgNf1.2 *t*-test | $P < 0.0001$ |
| | 21 weeks post-infection: sgTom vs. sgNf1.3 *t*-test | $P < 0.0001$ |
| | 21 weeks post-infection: sgNf. 1 vs. sgNf1.3 *t*-test | $P = 0.0133$ |
| | 21 weeks post-infection: sgNf1.2 vs. sgNf1.3 *t*-test | $P = 0.0180$ |
| Figure 1 (I) | 21 weeks post-infection: sgTom vs. sgNf1.1 *t*-test | $P < 0.0001$ |
| | 21 weeks post-infection: sgTom vs. sgNf1.2 *t*-test | $P < 0.0001$ |
| | 21 weeks post-infection: sgTom vs. sgNf1.3 *t*-test | $P < 0.0001$ |
| | 21 weeks post-infection: sgNf. 1 vs. sgNf1.3 *t*-test | $P = 0.0010$ |
| | 21 weeks post-infection: sgNf1.2 vs. sgNf1.3 *t*-test | $P < 0.0001$ |
| Figure 1 (J) | Tumor Grade 1 vs. Grade 3 *t*-test | $P = 0.0014$ |
| | Tumor Grade 1 vs. Grade 4 *t*-test | $P < 0.0001$ |
| | Tumor Grade 2 vs. Grade 3 *t*-test | $P = 0.0019$ |
| | Tumor Grade 2 vs. Grade 4 *t*-test | $P < 0.0001$ |
| Figure 1 (K) | 21 weeks post-infection: sgTom vs. sgNf1.1 *t*-test | $P < 0.0001$ |
| | 21 weeks post-infection: sgTom vs. sgNf1.2 *t*-test | $P < 0.0001$ |
| | 21 weeks post-infection: sgTom vs. sgNf1.3 *t*-test | $P < 0.0001$ |
| | 21 weeks post-infection: sgNf. 1 vs. sgNf1.3 *t*-test | $P = 0.0033$ |
| | 21 weeks post-infection: sgNf1.2 vs. sgNf1.3 *t*-test | $P = 0.0004$ |
| Figure 1 (L) | Tumor Grade 1 vs. Grade 3 *t*-test | $P = 0.0143$ |
| | Tumor Grade 1 vs. Grade 4 *t*-test | $P = 0.0003$ |
| | Tumor Grade 2 vs. Grade 3 *t*-test | $P = 0.0163$ |
| | Tumor Grade 2 vs. Grade 4 *t*-test | $P = 0.0154$ |
| Figure 2 (G) | *PSAT1* *t*-test | $P = 0.0011$ |
| | *AREG* *t*-test | $P = 0.0101$ |
| | *PEG10* *t*-test | $P = 0.0088$ |
| Figure 2 (H,I) | PDKN1 day 15 *t*-test | $P < 0.0001$ |
| | PDKN2 day 15 *t*-test | $P < 0.0001$ |
| Figure 2 (J,L) | SW1573 day 15 *t*-test | $P < 0.0001$ |
| | LKR10 day 15 *t*-test | $P < 0.0001$ |
| | LKR13 day 15 *t*-test | $P < 0.0001$ |

| | | |
|---|---|---|
| Figure 2 (M) | sgTom vs. sgNf1.3 *t*-test | $P < 0.0001$ |
| Figure 2 (O) | sgTom vs. sgNf1.3 *t*-test | $P = 0.0041$ |
| Figure 3 (A) | Glucose consumption: KP.1 vs. KP.1ΔNF1 *t*-test | $P = 0.0053$ |
| | Glucose consumption KP.2 vs. KP.2ΔNF1 *t*-test | $P = 0.0138$ |
| | lactate excretion: KP.1 vs. KP.1ΔNF1 *t*-test | $P = 0.0023$ |
| | lactate excretion KP.2 vs. KP.2ΔNF1 *t*-test | $P = 0.0040$ |
| Figure 3 (B) | Glutamine consumption KP.1 vs. KP.1ΔNF1 *t*-test | $P = 0.0012$ |
| | Glutamine consumption KP.2 vs. KP.2ΔNF1 *t*-test | $P = 0.0024$ |
| Figure 3 ( C) | Log GPNA of 5: KP.1 vs. KP.1ΔNF1 *t*-test | $P = 0.0032$ |
| | Log GPNA of 5KP.2 vs. KP.2ΔNF1 *t*-test | $P = 0.0177$ |
| Figure 3 (D) | Day 6: 2.0 mM Gln KPΔNF1 vs. 0.5 mM Gln KPΔNF1 *t*-test | $P = 0.0025$ |
| Figure 3 (I) | Day 6: sgTom KP.1 vs. sgPsat1 KP.1ΔNF1 *t*-test | $P = 0.0010$ |
| | Day 6:sgTom KP.2 vs. sgPsat1 KP.2ΔNF1 *t*-test | $P = 0.0061$ |
| Figure 3 (J-L) | PDKN1 Day 6: sgTom vs. sgPsat1 *t*-test | $P < 0.0001$ |
| | PDKN2 Day 6: sgTom vs. sgPsat1 *t*-test | $P < 0.0001$ |
| Figure 4 (B,C) | CB-839 (50 nM): KP.1 vs. KP.1ΔNF1 *t*-test | $P = 0.0003$ |
| | CB-839 (50 nM): KP.2 vs. KP.2ΔNF1 *t*-test | $P = 0.0012$ |
| | AOA (25 μM): KP.1 vs. KP.1ΔNF1 *t*-test | $P = 0.0007$ |
| | AOA (25 μM): KP.2 vs. KP.2ΔNF1 *t*-test | $P = 0.0031$ |
| Figure 4 (D) | KP.1ΔNF1 vs. KP.1ΔNF1 CB-839 *t*-test | $P = 0.0001$ |
| | KP.1ΔNF1 vs. KP.1ΔNF1 AOA *t*-test | $P = 0.0002$ |
| | KP.2ΔNF1 vs. KP.2ΔNF1 CB-839 *t*-test | $P = 0.0002$ |
| | KP.2ΔNF1 vs. KP.2ΔNF1 AOA *t*-test | $P = 0.0002$ |
| Figure 4 (E) | H2030 vs. H2030 CB-839 *t*-test | $P = 0.023$ |
| | H2030 vs. H2030 AOA *t*-test | $P = 0.023$ |
| | H2347 vs. H2347 CB-839 *t*-test | $P = 0.031$ |
| | H2347 vs. H2347 AOA *t*-test | $P = 0.033$ |
| Figure 5 (B,C) | CB-839 20 weeks post-infection: sgTom vehicle vs. sgNf1.3 vehicle *t*-test | $P < 0.0001$ |
| | CB-839 20 weeks post-infection: sgNf1.3 vehicle vs. sgNf1.3 CB-839 *t*-test | $P < 0.0001$ |
| | AOA 20 weeks post-infection: sgTom vehicle vs. sgNf1.3 vehicle *t*-test | $P < 0.0001$ |
| | AOA 20 weeks post-infection: sgNf1.3 vehicle vs. sgNf1.3 AOA *t*-test | $P < 0.0001$ |
| Figure 5 (D,E) | CB-839 20 weeks post-infection: sgTom vehicle vs. sgNf1.3 vehicle *t*-test | $P < 0.0001$ |
| | CB-839 20 weeks post-infection: sgNf1.3 vehicle vs. sgNf1.3 CB-839 *t*-test | $P < 0.0001$ |
| | AOA 20 weeks post-infection: sgTom vehicle vs. sgNf1.3 vehicle *t*-test | $P < 0.0001$ |
| | AOA 20 weeks post-infection: sgNf1.3 vehicle vs. sgNf1.3 AOA *t*-test | $P < 0.0001$ |
| Figure 6 (D) | Tumor mass: KPΔNF1 vehicle vs. KPΔNF1 CB-839 *t*-test | $P = 0.560$ |
| | Tumor mass: KPΔNF1 vehicle vs. KPΔNF1 AOA | $P = 0.1046$ |

| | | |
|---|---|---|
| Figure S1 (C,D) | Day 25: KP.1 vs. KP.1ΔNF1 *t*-test | $P < 0.0001$ |
| | Day 25: KP.2 vs. KP.2ΔNF1 *t*-test | $P < 0.0001$ |
| Figure S1 (E,F) | KP.1 vs. KP.1ΔNF1 *t*-test | $P < 0.0001$ |
| | KP.2 vs. KP.2ΔNF1 *t*-test | $P < 0.0001$ |
| Figure S1 (G,H) | KP.1 vs. KP.1ΔNF1 *t*-test | $P < 0.0001$ |
| | KP.2 vs. KP.2ΔNF1 *t*-test | $P < 0.0001$ |
| Figure S1 (I,J) | Day 30: KP.1 vs. KP.1ΔNF1 *t*-test | $P < 0.0001$ |
| | Day 30: KP.2 vs. KP.2ΔNF1 *t*-test | $P < 0.0001$ |
| Figure S1 (K) | Day 10: KP.1 vs. KP.1ΔNF1 *t*-test | $P = 0.0029$ |
| | Day 10: KP.2 vs. KP.2ΔNF1 *t*-test | $P = 0.0034$ |
| Figure S2 (A) | PDKN1 *t*-test | $P = 0.1065$ |
| | PDKN2 *t*-test | $P = 0.1390$ |
| | SW1573 *t*-test | $P = 0.1156$ |
| | LKR10 *t*-test | $P = 0.0610$ |
| | LKR13 *t*-test | $P = 0.1223$ |
| Figure S2 (B) | Nf1 *t*-test | $P = 0.0004$ |
| | p-Fak1 *t*-test | $P = 0.0008$ |
| Figure S2 (C) | Nf1 *t*-test | $P = 0.0020$ |
| | p-Fak1 *t*-test | $P = 0.0028$ |
| Figure S2 (D) | Nf1 *t*-test | $P = 0.0007$ |
| | p-Fak1 *t*-test | $P = 0.0002$ |
| Figure S2 (E) | Nf1 *t*-test | $P = 0.0127$ |
| | p-Fak1 *t*-test | $P = 0.0001$ |
| Figure S2 (F) | Nf1 *t*-test | $P = 0.0040$ |
| | p-Fak1 *t*-test | $P = 0.0011$ |
| Figure S3 (B) | *Psat1*: KP.1 vs. KP.1ΔNF1 *t*-test | $P = 0.0193$ |
| | *Psat1*: KP.2 vs. KP.2ΔNF1 *t*-test | $P = 0.0144$ |
| | *Psat1*: KP.1 vs. KP.1ΔFAK1 *t*-test | $P = 0.0018$ |
| | *Psat1*: KP.2 vs. KP.2ΔFAK1 *t*-test | $P = 0.0059$ |
| | *Areg*: KP.1 vs. KP.1ΔNF1 *t*-test | $P = 0.010$ |
| | *Areg*: KP.2 vs. KP.2ΔNF1 *t*-test | $P = 0.0073$ |
| | *Areg*: KP.1 vs. KP.1ΔFAK1 *t*-test | $P = 0.0112$ |
| | *Areg*: KP.2 vs. KP.2ΔFAK1 *t*-test | $P = 0.0052$ |
| | *Peg10*: KP.1 vs. KP.1ΔNF1 *t*-test | $P = 0.0013$ |
| | *Peg10*: KP.2 vs. KP.2ΔNF1 *t*-test | $P < 0.0001$ |
| | *Peg10*: KP.1 vs. KP.1ΔFAK1 *t*-test | $P = 0.0079$ |
| | *Peg10*: KP.2 vs. KP.2ΔFAK1 *t*-test | $P = 0.0126$ |
| Figure S3 (D-F) | *Psat1*: KP.1 vs. KP.1 + PIP2 *t*-test | $P = 0.0034$ |
| | *Psat1*: KP.2 vs. KP.2 + PIP2 *t*-test | $P = 0.0080$ |
| | *Psat1*: KP.1ΔNF1 vs. KP.1ΔNF1 + PIP2 *t*-test | $P = 0.0169$ |
| | *Psat1*: KP.2ΔNF1 vs. KP.2ΔNF1 + PIP2 *t*-test | $P = 0.0206$ |
| | *Psat1*: KP.1 vs. KP.1ΔNF1 *t*-test | $P = 0.0066$ |
| | *Psat1*: KP.1 + PIP2 vs. KP.1ΔNF1 + PIP2 *t*-test | $P = 0.0238$ |
| | *Psat1*: KP.2 vs. KP.2ΔNF1 *t*-test | $P = 0.0003$ |
| | *Psat1*: KP.2 + PIP2 vs. KP.2ΔNF1 + PIP2 *t*-test | $P = 0.0138$ |

| | | |
|---|---|---|
| | *Psat1*: KP.1 vs. KP.1ΔFAK1 *t*-test | $P = 0.0105$ |
| | *Psat1*: KP.1 + PIP2 vs. KP.1ΔFAK1 + PIP2 *t*-test | $P = 0.0033$ |
| | *Psat1*: KP.2 vs. KP.2ΔFAK1 *t*-test | $P = 0.0076$ |
| | *Psat1*: KP.2 + PIP2 vs. KP.2ΔFAK1 + PIP2 *t*-test | $P = 0.0062$ |
| | *Areg*: KP.1 vs. KP.1 + PIP2 *t*-test | $P = 0.0020$ |
| | *Areg*: KP.2 vs. KP.2 + PIP2 *t*-test | $P < 0.0001$ |
| | *Areg*: KP.1ΔNF1 vs. KP.1ΔNF1 + PIP2 *t*-test | $P = 0.0076$ |
| | *Areg*: KP.2ΔNF1 vs. KP.2ΔNF1 + PIP2 *t*-test | $P = 0.0031$ |
| | *Areg*: KP.1 vs. KP.1ΔNF1 *t*-test | $P = 0.0124$ |
| | *Areg*: KP.1 + PIP2 vs. KP.1ΔNF1 + PIP2 *t*-test | $P = 0.0174$ |
| | *Areg*: KP.2 vs. KP.2ΔNF1 *t*-test | $P = 0.0180$ |
| | *Areg*: KP.2 + PIP2 vs. KP.2ΔNF1 + PIP2 *t*-test | $P = 0.010$ |
| | *Areg*: KP.1 vs. KP.1ΔFAK1 *t*-test | $P = 0.0158$ |
| | *Areg*: KP.1 + PIP2 vs. KP.1ΔFAK1 + PIP2 *t*-test | $P = 0.0020$ |
| | *Areg*: KP.2 vs. KP.2ΔFAK1 *t*-test | $P = 0.0061$ |
| | *Areg*: KP.2 + PIP2 vs. KP.2ΔFAK1 + PIP2 *t*-test | $P = 0.0003$ |
| | *Peg10*: KP.1 vs. KP.1 + PIP2 *t*-test | $P = 0.0481$ |
| | *Peg10*: KP.2 vs. KP.2 + PIP2 *t*-test | $P = 0.0715$ |
| | *Peg10*: KP.1ΔNF1 vs. KP.1ΔNF1 + PIP2 *t*-test | $P = 0.0059$ |
| | *Peg10*: KP.2ΔNF1 vs. KP.2ΔNF1 + PIP2 *t*-test | $P = 0.1022$ |
| | *Peg10*: KP.1 vs. KP.1ΔNF1 *t*-test | $P = 0.0548$ |
| | *Peg10*: KP.1 + PIP2 vs. KP.1ΔNF1 + PIP2 *t*-test | $P = 0.0057$ |
| | *Peg10*: KP.2 vs. KP.2ΔNF1 *t*-test | $P = 0.0177$ |
| | *Peg10*: KP.2 + PIP2 vs. KP.2ΔNF1 + PIP2 *t*-test | $P = 0.0781$ |
| | *Peg10*: KP.1 vs. KP.1ΔFAK1 *t*-test | $P = 0.0099$ |
| | *Peg10*: KP.1 + PIP2 vs. KP.1ΔFAK1 + PIP2 *t*-test | $P = 0.0473$ |
| | *Peg10*: KP.2 vs. KP.2ΔFAK1 *t*-test | $P = 0.0872$ |
| | *Peg10*: KP.2 + PIP2 vs. KP.2ΔFAK1 + PIP2 *t*-test | $P = 0.0690$ |
| Figure S4 (A) | KP.1ΔNF1 vs. KP.1ΔNF1 + 2DG *t*-test | $P = 0.0005$ |
| | KP.2ΔNF1 vs. KP.2ΔNF1 + 2DG *t*-test | $P = 0.007$ |
| Figure S4 (B) | M+3 citrate: KP.1 vs. KP.1ΔNF1 *t*-test | $P = 0.020$ |
| Figure S4 (C,D) | KP.1 vs. KP.1ΔNF1 citrate *t*-test | $P = 0.0268$ |
| | KP.1 vs. KP.1ΔNF1 isocitrate *t*-test | $P = 0.0272$ |
| | KP.1 vs. KP.1ΔNF1 aKG *t*-test | $P = 0.0002$ |
| | KP.1 vs. KP.1ΔNF1 succinate *t*-test | $P = 0.0005$ |
| | KP.1 vs. KP.1ΔNF1 fumarate *t*-test | $P = 0.0033$ |
| | KP.1 vs. KP.1ΔNF1 malate *t*-test | $P = 0.0116$ |
| | KP.1 vs. KP.1ΔNF1 aspartate *t*-test | $P = 0.0019$ |
| Figure S5 (A,B) | CB-839: PDK vs. PDKN1 *t*-test | $P < 0.0001$ |
| | CB-839: PDK vs. PDKN2 *t*-test | $P = 0.0066$ |
| | AOA: PDK vs. PDKN1 *t*-test | $P < 0.0001$ |
| | AOA: PDK vs. PDKN2 *t*-test | $P = 0.0005$ |
| Figure S5 (C-E) | KP.1ΔNF1 vs. KP.1ΔNF1 + CB-839 *t*-test | $P = 0.0031$ |
| | KP.1ΔNF1 + CB-839 vs. KP.1ΔNF1 + CB-839 + DMG *t*-test | $P = 0.0190$ |
| | KP.1ΔNF1 vs. KP.1ΔNF1 + CB-839 *t*-test | $P = 0.0031$ |

| | | |
|---|---|---|
| | KP.1ΔNF1 + CB-839 vs. KP.1ΔNF1 + CB-839 + pyruvate *t*-test | *P* = 0.0182 |
| | KP.1ΔNF1 vs. KP.1ΔNF1 + CB-839 *t*-test | *P* = 0.0031 |
| | KP.1ΔNF1 + CB-839 vs. KP.1ΔNF1 + CB-839 + glutamate *t*-test | *P* = 0.0058 |
| Figure S5 (F-H) | KP.1ΔNF1 vs. KP.1ΔNF1 + AOA *t*-test | *P* = 0.0024 |
| | KP.1ΔNF1 + AOA vs. KP.1ΔNF1 + CB-839 + DMG *t*-test | *P* = 0.0329 |
| | KP.1ΔNF1 vs. KP.1ΔNF1 + AOA *t*-test | *P* = 0.0024 |
| | KP.1ΔNF1 + AOA vs. KP.1ΔNF1 + AOA + pyruvate *t*-test | *P* = 0.0386 |
| | KP.1ΔNF1 vs. KP.1ΔNF1 + AOA *t*-test | *P* = 0.0024 |
| | KP.1ΔNF1 + AOA vs. KP.1ΔNF1 + CB-839 + glutamate *t*-test | *P* = 0.0038 |
| Figure S6 (B-D) | *Psat1*: KP.1 vs. KP.1 + PIP2 *t*-test | *P* = 0.0002 |
| | *Psat1*: KP.1 vs. KP.1 + PIP2 + Dox *t*-test | *P* = 0.0012 |
| | *Psat1*: KP.1 + PIP2 vs. KP.1 + PIP2 + Dox *t*-test | *P* = 0.0064 |
| | *Psat1*: KP.1 + Dox vs. KP.1 + PIP2 + Dox *t*-test | *P* = 0.0011 |
| | *Psat1*: KP.2 vs. KP.2 + PIP2 *t*-test | *P* = 0.0001 |
| | *Psat1*: KP.2 vs. KP.2 + PIP2 + Dox *t*-test | *P* < 0.0001 |
| | *Psat1*: KP.2 + PIP2 vs. KP.2 + PIP2 + Dox *t*-test | *P* = 0.0010 |
| | *Psat1*: KP.2 + Dox vs. KP.2 + PIP2 + Dox *t*-test | *P* = 0.0001 |
| | *Areg*: KP.1 vs. KP.1 + PIP2 *t*-test | *P* = 0.0014 |
| | *Areg*: KP.1 vs. KP.1 + PIP2 + Dox *t*-test | *P* = 0.0049 |
| | *Areg*: KP.1 + PIP2 vs. KP.1 + PIP2 + Dox *t*-test | *P* = 0.0297 |
| | *Areg*: KP.1 + Dox vs. KP.1 + PIP2 + Dox *t*-test | *P* = 0.0049 |
| | *Areg*: KP.2 vs. KP.2 + PIP2 *t*-test | *P* = 0.0002 |
| | *Areg*: KP.2 vs. KP.2 + PIP2 + Dox *t*-test | *P* = 0.0001 |
| | *Areg*: KP.2 + PIP2 vs. KP.2 + PIP2 + Dox *t*-test | *P* = 0.0001 |
| | *Areg*: KP.2 + Dox vs. KP.2 + PIP2 + Dox *t*-test | *P* = 0.0001 |
| | *Peg10*: KP.1 vs. KP.1 + PIP2 *t*-test | *P* < 0.0001 |
| | *Peg10*: KP.1 vs. KP.1 + PIP2 + Dox *t*-test | *P* = 0.0016 |
| | *Peg10*: KP.1 + PIP2 vs. KP.1 + PIP2 + Dox *t*-test | *P* = 0.0127 |
| | *Peg10*: KP.1 + Dox vs. KP.1 + PIP2 + Dox *t*-test | *P* = 0.0016 |
| | *Peg10*: KP.2 vs. KP.2 + PIP2 *t*-test | *P* = 0.0004 |
| | *Peg10*: KP.2 vs. KP.2 + PIP2 + Dox *t*-test | *P* < 0.0001 |
| | *Peg10*: KP.2 + PIP2 vs. KP.2 + PIP2 + Dox *t*-test | *P* = 0.0002 |
| | *Peg10*: KP.2 + Dox vs. KP.2 + PIP2 + Dox *t*-test | *P* < 0.0001 |
| Figure S6 (E) | KP-ix vs. KP-ix + CB-839 *t*-test | *P* = 0.0053 |
| | KP-ix vs. KP-ix + CB-839 + Dox *t*-test | *P* < 0.0001 |
| | KP-ix + CB-839 vs. KP-ix + CB-839 + Dox *t*-test | *P* = 0.0002 |
| | KP-ix + Dox vs. KP-ix + CB-839 + Dox *t*-test | *P* < 0.0001 |
| Figure S6 (F) | KP-ix vs. KP-ix + AOA *t*-test | *P* = 0.0168 |
| | KP-ix vs. KP-ix + AOA + Dox *t*-test | *P* = 0.0004 |
| | KP-ix + AOA vs. KP-ix + AOA + Dox *t*-test | *P* = 0.0017 |
| | KP-ix + Dox vs. KP-ix + AOA + Dox *t*-test | *P* < 0.0001 |
| Figure S6 (H-J) | *Psat1*: KP.1ΔNF1 PGK Ctrl vs. KP.1ΔNF1 PGK Nf1 *t*-test | *P* = 0.0023 |
| | *Psat1*:KP.2ΔNF1 PGK Ctrl vs. KP.2ΔNF1 PGK Nf1 *t*-test | *P* = 0.0002 |
| | *Areg*: KP.1ΔNF1 PGK Ctrl vs. KP.1ΔNF1 PGK Nf1 *t*-test | *P* < 0.0001 |

| | | |
|---|---|---|
| | *Areg*: KP.2ΔNF1 PGK Ctrl vs. KP.2ΔNF1 PGK Nf1 *t*-test | $P < 0.0001$ |
| | *Peg10*: KP.1ΔNF1 PGK Ctrl vs. KP.1ΔNF1 PGK Nf1 *t*-test | $P < 0.0001$ |
| | *Peg10*: KP.2ΔNF1 PGK Ctrl vs. KP.2ΔNF1 PGK Nf1 *t*-test | $P < 0.0001$ |
| Figure S6 (K) | Day 25: KP.1ΔNF1 PGK Ctrl vs. KP.1ΔNF1 PGK Nf1 *t*-test | $P = 0.0001$ |
| Figure S6 (L,M) | KP.1ΔNF1 PGK Ctrl vehicle vs. KP.1ΔNF1 PGK Ctrl CB-839 *t*-test | $P = 0.0083$ |
| | KP.1ΔNF1 PGK Ctrl CB-839 vs. KP.1ΔNF1 PGK Nf1 CB-839 *t*-test | $P = 0.0010$ |
| | KP.1ΔNF1 PGK Ctrl vehicle vs. KP.1ΔNF1 PGK Ctrl AOA *t*-test | $P = 0.0007$ |
| | KP.1ΔNF1 PGK Ctrl AOA vs. KP.1ΔNF1 PGK Nf1 AOA *t*-test | $P = 0.0016$ |
| Figure S7 (A,B) | Day 27: KPΔNF1 vehicle vs. KPΔNF1 CB-839 *t*-test | $P < 0.0001$ |
| | Day 27: KPΔNF1 vehicle vs. KPΔNF1 AOA | $P < 0.0001$ |

## APPENDIX FIGURE LEGENDS

**Appendix Figure S1. Nf1-Silenced Xenograft Tumors Display Selective Growth Advantage**

(A) Schematic of the generation of the indicated cell lines (n = two cell lines per genotype). KP.1 and KP.2 cells were electroporated with the pX458 plasmid containing sgTom, sgNf1.3, or sgFak1.1 sgRNAs and then sorted by GFP expression.

(B) Western blotting analysis of indicated cell lines with Gapdh used as a loading control.

(C, D) Subcutaneous tumor volumes after orthotopic transplantation with (C) KP.1 or KP.1ΔNF1 cells and (D) KP.2 and KP.2ΔNF1 cells injected into nude mice (n = 50 tumors per cell line).

(E, F) Resulting final subcutaneous tumor masses for (E) KP.1 or KP.1ΔNF1 cells and (F) KP.2 or KP.2ΔNF1 cells (n = 50 tumors per cell line).

(G, H) Quantification of Ki-67-positive cell % in extracted subcutaneous (G) KP.1 or KP.1ΔNF1 tumors and (H) KP.2 or KP.2ΔNF1 cells (n = 50 tumors per cell line).

(I, J) Quantification of orthotopic tumor growth by photon flux luminescence after transplantation with (I) KP.1 or KP.1ΔNF1 cells and (J) KP.2 or KP.2ΔNF1 cells transduced with a luciferase vector (n = 50 tumors per cell line). Relative photon flux for each animal was calculated by normalizing all time points to baseline measurements.

(K) Cumulative population doublings of indicated cell lines in vitro (n = 4 biological replicates).

*P*-values are reported in Appendix Table S3. For bar charts, data presented as means with error bars representing standard deviations (SDs). For boxplots, whiskers indicate the minimum and maximum values, the upper and lower perimeters represent the first and third quartiles, the midline represents the median value, and the x symbol represents the mean.

**Appendix Figure S2. Full Experimental Data Supporting Figure 2**

(A) Quantitation of p53 protein expression depicted in Figure 2A.

(B) Quantitation of Nf1, p-Fak1, and Fak1 expression depicted in Figure 2B.

(C) Quantitation of Nf1, p-Fak1, and Fak1 expression depicted in Figure 2C.

(D) Quantitation of Nf1, p-Fak1, and Fak1 expression depicted in Figure 2D.

(E) Quantitation of Nf1, p-Fak1, and Fak1 expression depicted in Figure 2E.

(F) Quantitation of Nf1, p-Fak1, and Fak1 expression depicted in Figure 2F.

*P*-values are reported in Appendix Table S3. Data presented as means with error bars representing standard deviations (SDs).

**Appendix Figure S3. Nf1 Silencing Induces Fak1 Activation**

(A) Western blotting analysis of indicated cell lines with Gapdh used as a loading control. Note that p-Fak1 and Psat1 upregulation only occur in the KPΔNF1 clones.

(B) qRT-PCR analyzing mRNA expression of the Fak1 target genes -- *Psat1*, *Areg*, and *Peg10* -- in the indicated cell lines. The *y*-axis depicts the fold-change relative to KP1 baseline expression for each gene.

(C) Western blotting analysis of CRISPR-targeted KP clones with or without the Fak1 activator phosphatidylinsositol-4,5-bis-phosphate (PIP2; 10 µM for 6 h). Gapdh was used as a loading control. Note that p-Fak1 upregulation only occurs in PIP2-stimulated KP clones and is further upregulated in PIP2-stimulated KPΔNF1 clones.

(D-F) qRT-PCR analyzing mRNA expression of the Fak1 target genes -- *Psat1*, *Areg*, and *Peg10* -- in the KP cell lines treated with PIP2 (10 µM for 6 h). The *y*-axis depicts the fold-change relative to KP1-PIP2 baseline expression for each gene.

*P*-values are reported in Appendix Table S3. Data presented as means with error bars representing standard deviations (SDs).

**Appendix Figure S4. Nf1-Silenced Cells are More Glycolytic**

(A) Relative viability of KP and KPΔNF1 cells cultured with or without 5 mM 2DG for 72 h by trypan blue exclusion assay (n = 4 biological replicates).

(B) Schematic of glucose-derived carbon flux into the Krebs cycle via pyruvate dehydrogenase (M+2 citrate) or pyruvate carboxylase (M+3 citrate). Carbons (colored circles) are color-coded to match their source.

(C, D) Cells were cultured in RPMI with [U-13C]-L-glucose for 24 hours (n = 4 biological replicates). The graphs display (C) the % enrichment of the named isotopomers in the total citrate pool normalized by cell count and (D) the % mole enrichment of glucose-derived $^{13}$C carbon calculated for each Krebs cycle intermediate normalized by cell count.

*P*-values are reported in Appendix Table S3. Data presented as means with error bars representing standard deviations (SDs).

**Appendix Figure S5. Glutaminase and Aminotransferase Inhibition Specifically Inhibits Nf1-Mutant Cell Proliferation**

(A, B) Viability of patient-derived PDK, PDKN1, and PDKN2 cell lines treated with (A) 18 nM CB-839 or (B) 6 µM AOA for 72 h. PDK cell line is the same control group for (A) and (B).

(C-E) Relative growth of KP and KPΔNF1 cells incubated with and without CB-839 (18 nM) in the presence or absence of (C) permeable α-ketoglutarate (DMG), (D) pyruvate, or (E) glutamate. KP and KPΔNF1 cells are the same control groups for (C), (D), and (E).

(F-H) Relative growth of KP and KPΔNF1 cells incubated with and without AOA (6 µM) in the presence or absence of (F) DMG, (G) pyruvate, or (H) glutamate. KP and KPΔNF1 cells are the same control groups for (F), (G), and (H).

*P*-values are reported in Appendix Table S3. Data presented as means with error bars representing standard deviations (SDs).

**Appendix Figure S6. Fak1 Activity Required for Sensitivity to Glutaminase and Aminotransferase Inhibition**

Two independent KP cell lines were constructed with a doxycycline (DOX)-inducible HA-tagged gain-of-function (GOF)-Fak1 cDNA (KP-ix). As indicated, the GOF-Fak1 cDNA was induced by DOX for 72 h and then treated with PIP2 (10 μM for 6 h).

(A) Western blotting analysis of HA expression and Fak1 activation in the KP-ix cell lines.

(B-D) qRT-PCR analyzing mRNA expression of the Fak1 target genes – (B) *Psat1*, (C) *Areg*, and (D) *Peg10* -- in the KP-ix cell lines. The *y*-axis depicts the fold-change relative to the control KP1-ix cell line for each target gene (n = 4 biological replicates per target gene per cell line).

(E) Relative viability of KP-ix cells with a DOX-inducible GOF-Fak1 cDNA treated with control, DOX, CB-839 (18 nM), or DOX+CB-839 for 72 h (n = 4 biological replicates).

(G) Western blotting analysis of Fak1 activation in KPΔNF1 cells transduced with Nf1 or control cDNAs.

(H-J) qRT-PCR analyzing mRNA expression of the Fak1 target genes – (H) *Psat1*, (I) *Areg*, and (J) *Peg10* -- in the KPΔNF1 cell line transduced with control or Nf1 cDNAs. The *y*-axis depicts the fold-change relative to the control KP.1ΔNF1 cell line for each target gene (n = 4 biological replicates per target gene per cell line).

(K) Subcutaneous tumor volumes of KPΔNF1 cells transduced with control or Nf1 cDNAs.

(L, M) Relative cell growth of KP and KPΔNF1 cell lines transduced with control or Nf1 cDNAs with or without CB-839 (18 nM) or AOA (6 μM) for 72 h. All data points are relative to each genotype’s vehicle-treated control (n = 4 biological replicates per treatment per cell line).

*P*-values are reported in Appendix Table S3. Data presented as means with error bars representing standard deviations (SDs).

**Appendix Figure S7. Full Experimental Data Supporting Figure 6**

(A, B) Subcutaneous tumor volumes of KP and KPΔNF1 cells treated with vehicle, CB-839, or AOA starting from day 12 (arrow indicating treatment start) measured until day 27 (n = 6 tumors per cell line per treatment). Vehicle-treated KP and KPΔNF1 cells are the same control groups for (A) and (B).

(C, D) Orthotopic growth measurements by photon flux luminescence of KP and KPΔNF1 cells treated with vehicle, CB-839, or AOA starting from day 12 (arrow indicating treatment start) measured until day 27 (n = 24 mice per cell line per treatment). Vehicle-treated KP and KPΔNF1 cells are the same control groups for (C) and (D). Relative photon flux for each animal was calculated by normalizing all time points to baseline measurements at day 10 post-transplantation.

(E, F) Subcutaneous tumor volumes of KP-ix cells harboring doxycycline (DOX)-inducible gain-of-function (GOF)-Fak1 cDNA treated with vehicle, CB-839, or AOA with or without DOX (n = 36 mice per treatment group). Vehicle-treated KP-ix cells are the same control groups for (E) and (F).

(G-I) Subcutaneous tumor volumes of the patient-derived *NF1*-mutant (PDKN1 and PDKN2) and *NF1*-WT (PDK) LUAD cell lines treated with vehicle, CB-839, or AOA starting from day 12 (arrow indicating treatment start) measured until day 27 (n = 6 tumors per cell line per treatment).

(J-L) Orthotopic growth measurements by photon flux luminescence of the patient-derived *NF1*-mutant (PDKN1 and PDKN2) and *NF1*-WT (PDK) LUAD cell lines treated with vehicle, CB-839, or AOA starting from day 12 (arrow indicating treatment start) measured until day 27 (n = 12 mice per cell line per treatment). Relative photon flux for each animal was calculated by normalizing all time points to baseline measurements at day 10 post-transplantation.

*P*-values are reported in Appendix Table S3. Data presented as means with error bars representing standard deviations (SDs).

# APPENDIX FIGURES

## Appendix Figure S1

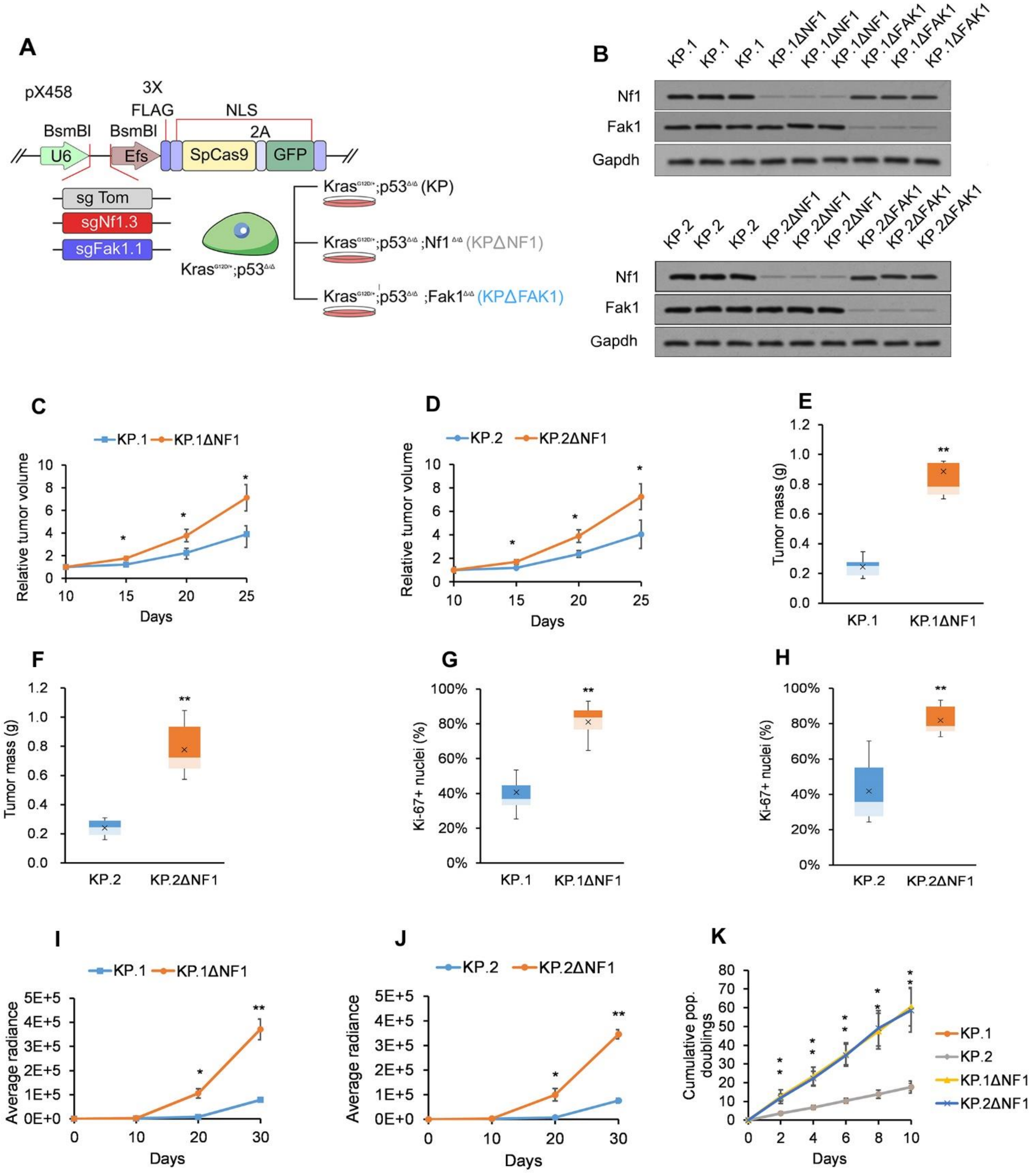

# Appendix Figure S2

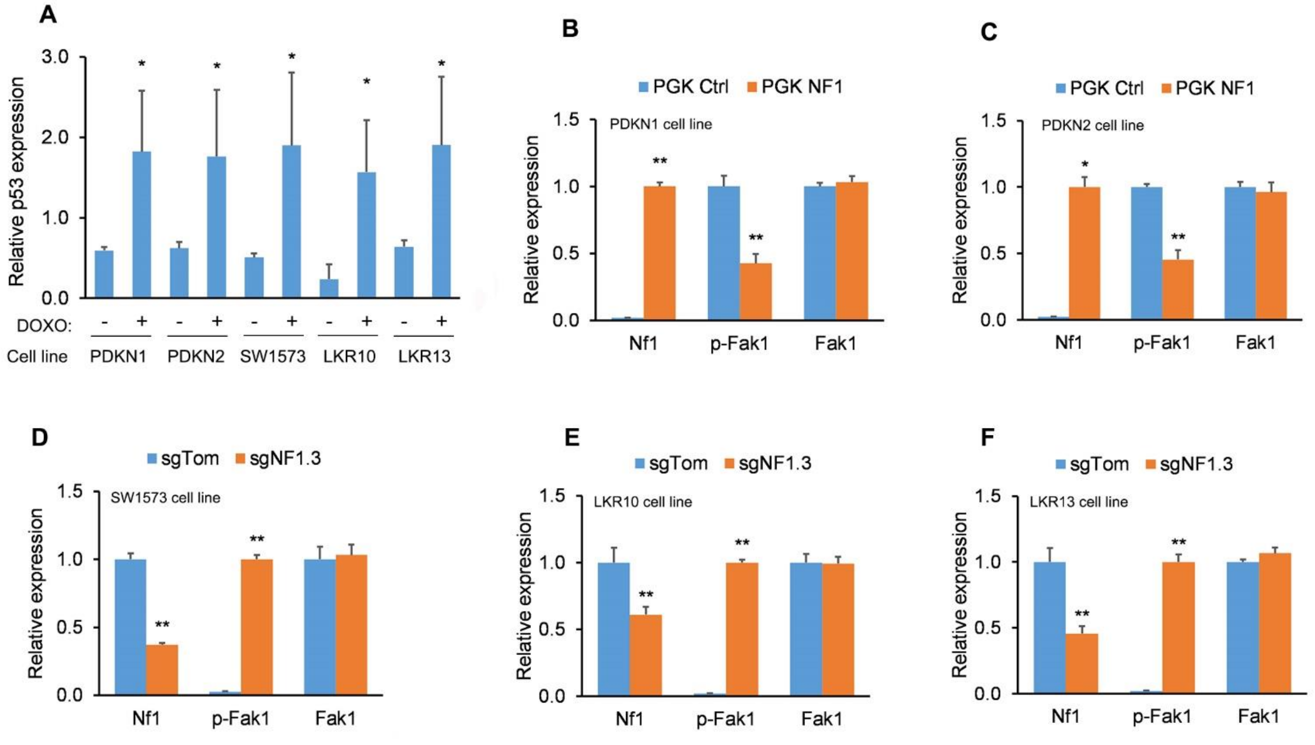

**Appendix Figure S3**

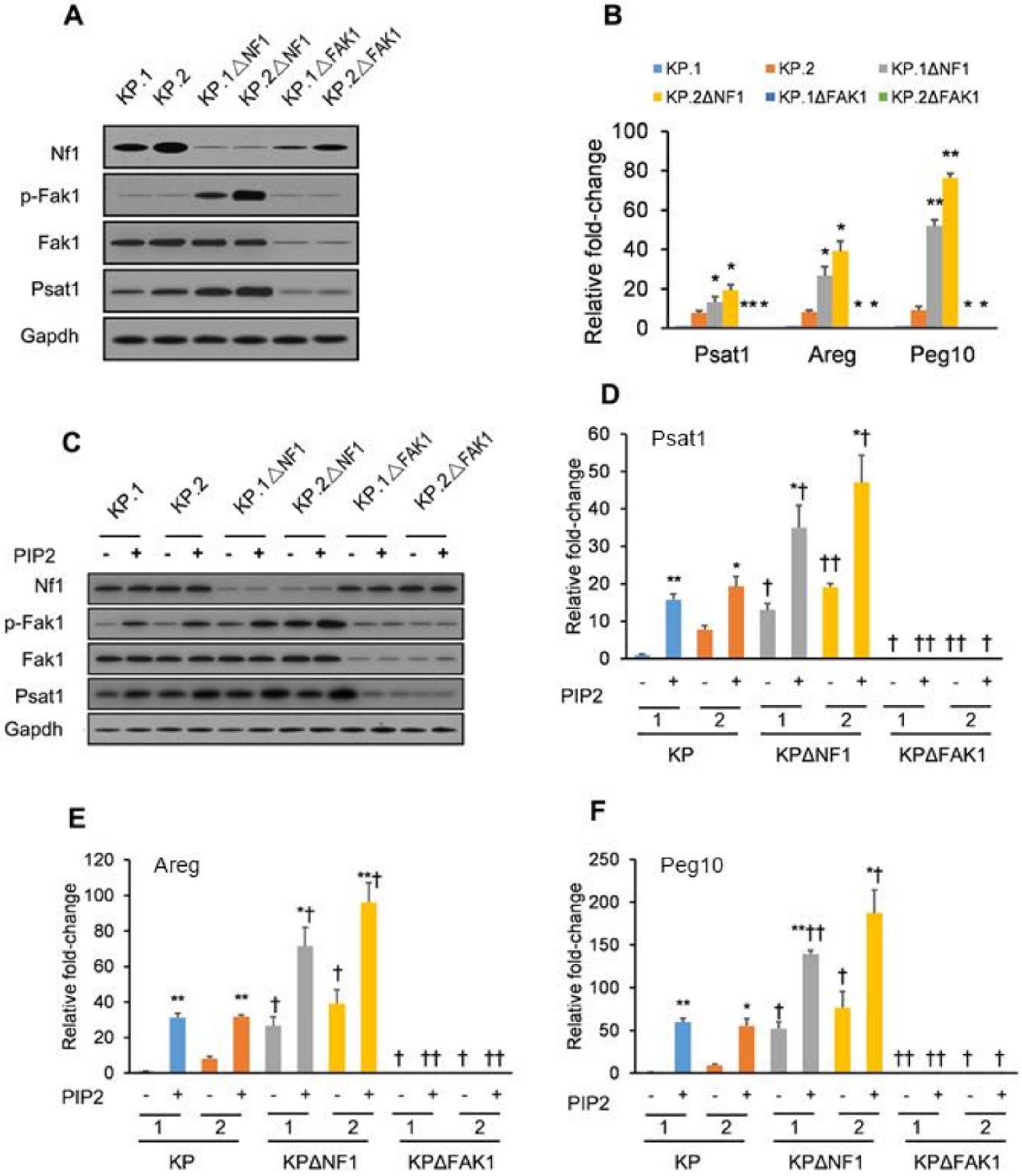

**Appendix Figure S4**

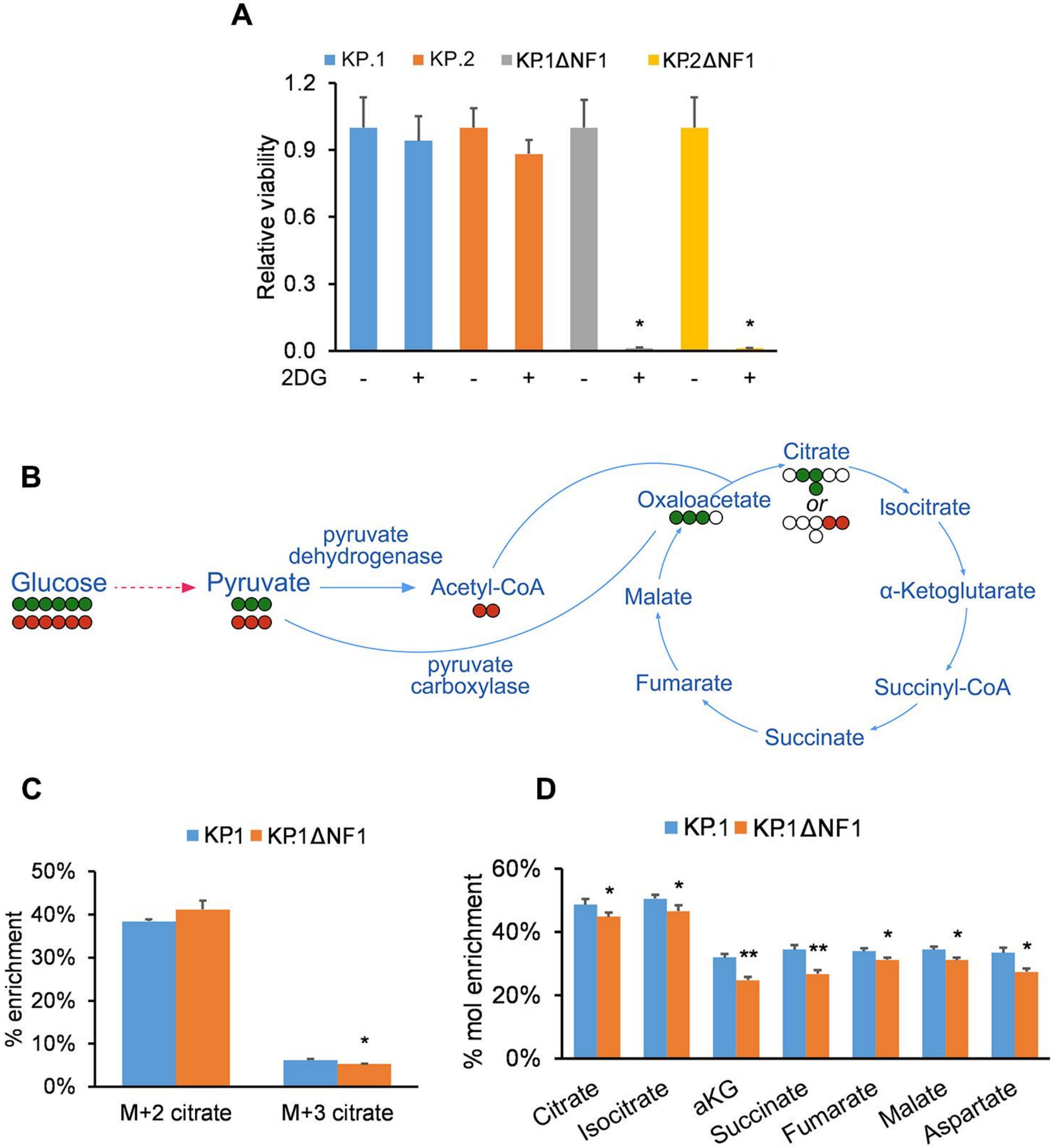

**Appendix Figure S5**

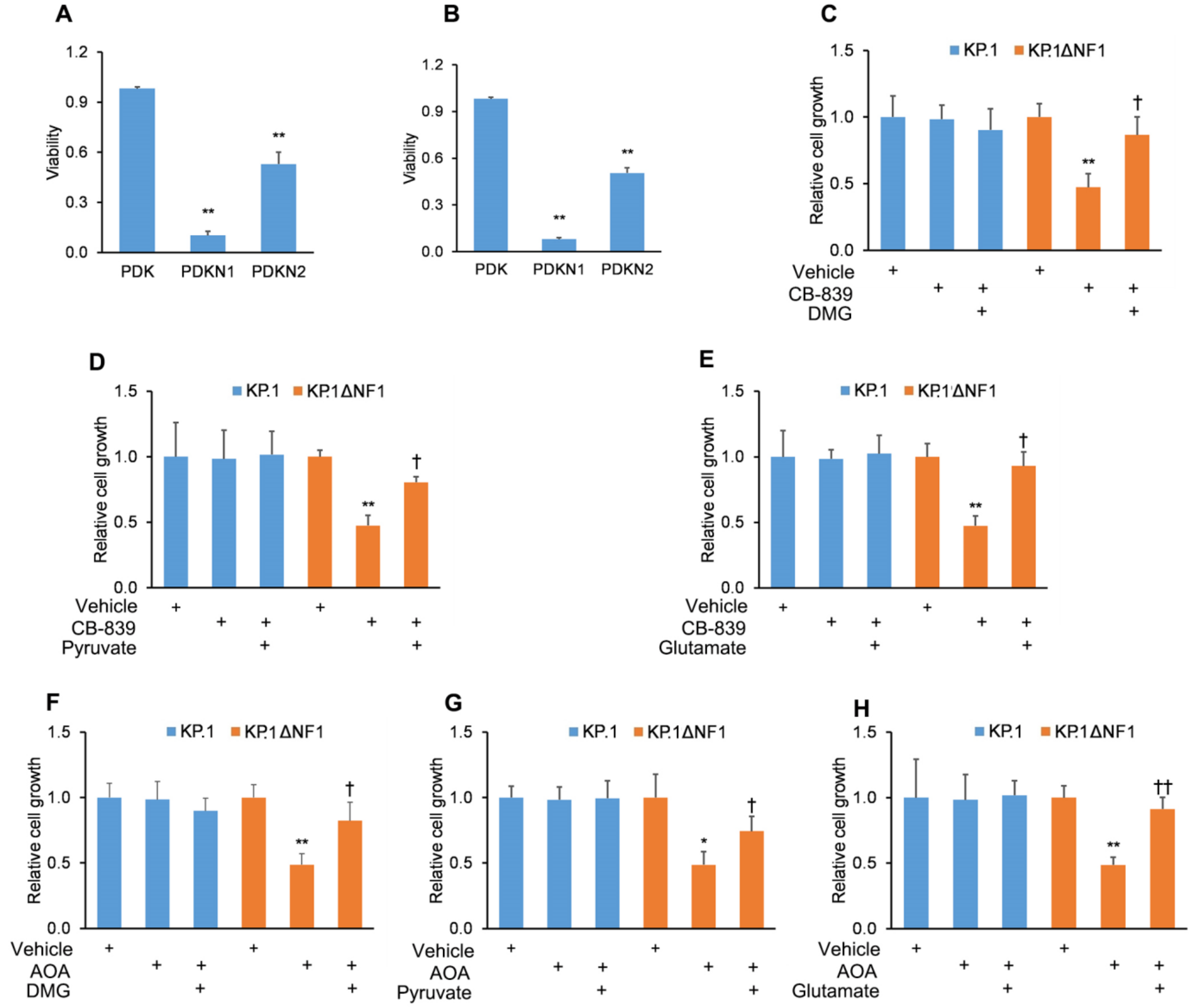

## Appendix Figure S6

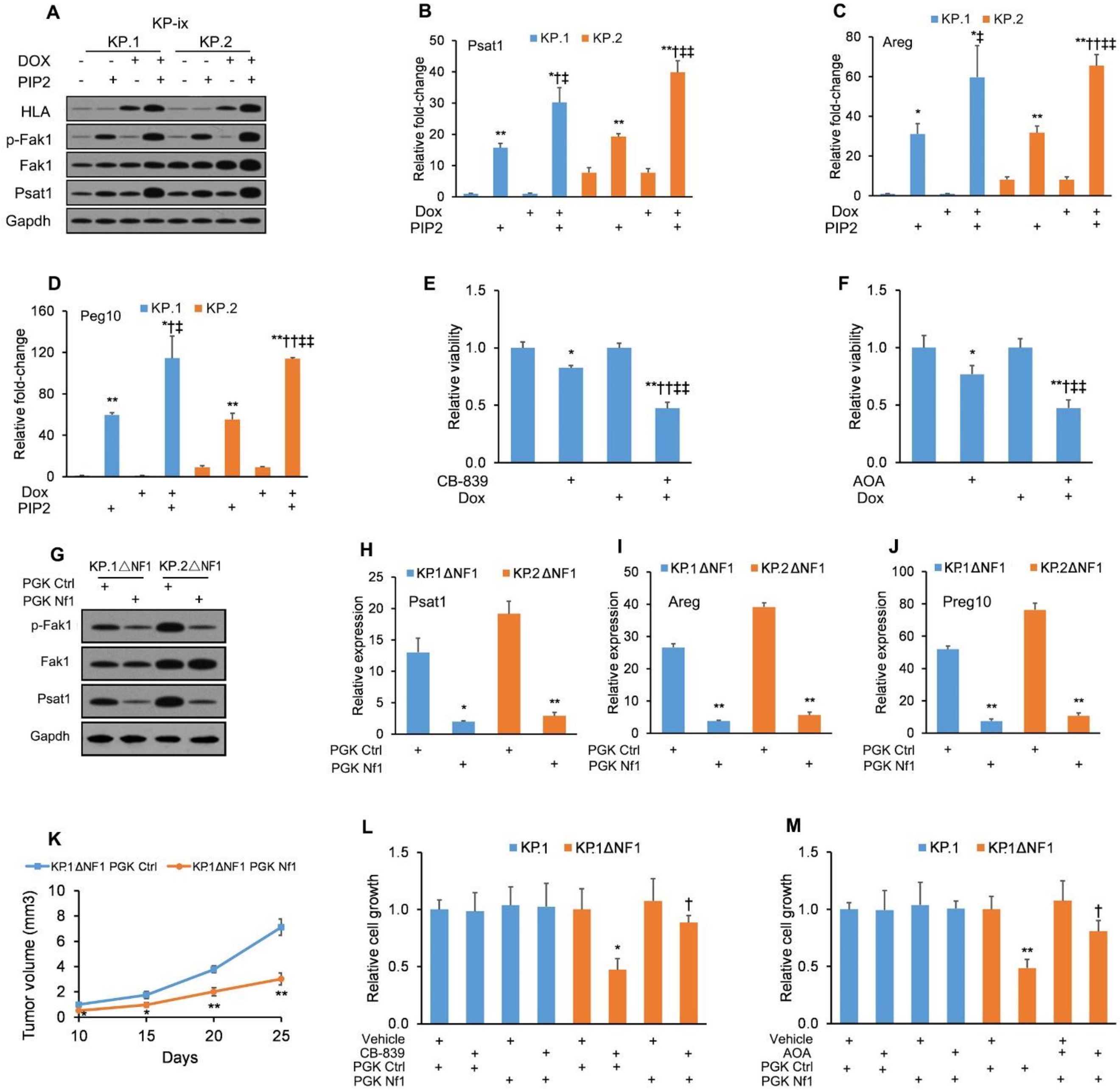

# Appendix Figure S7

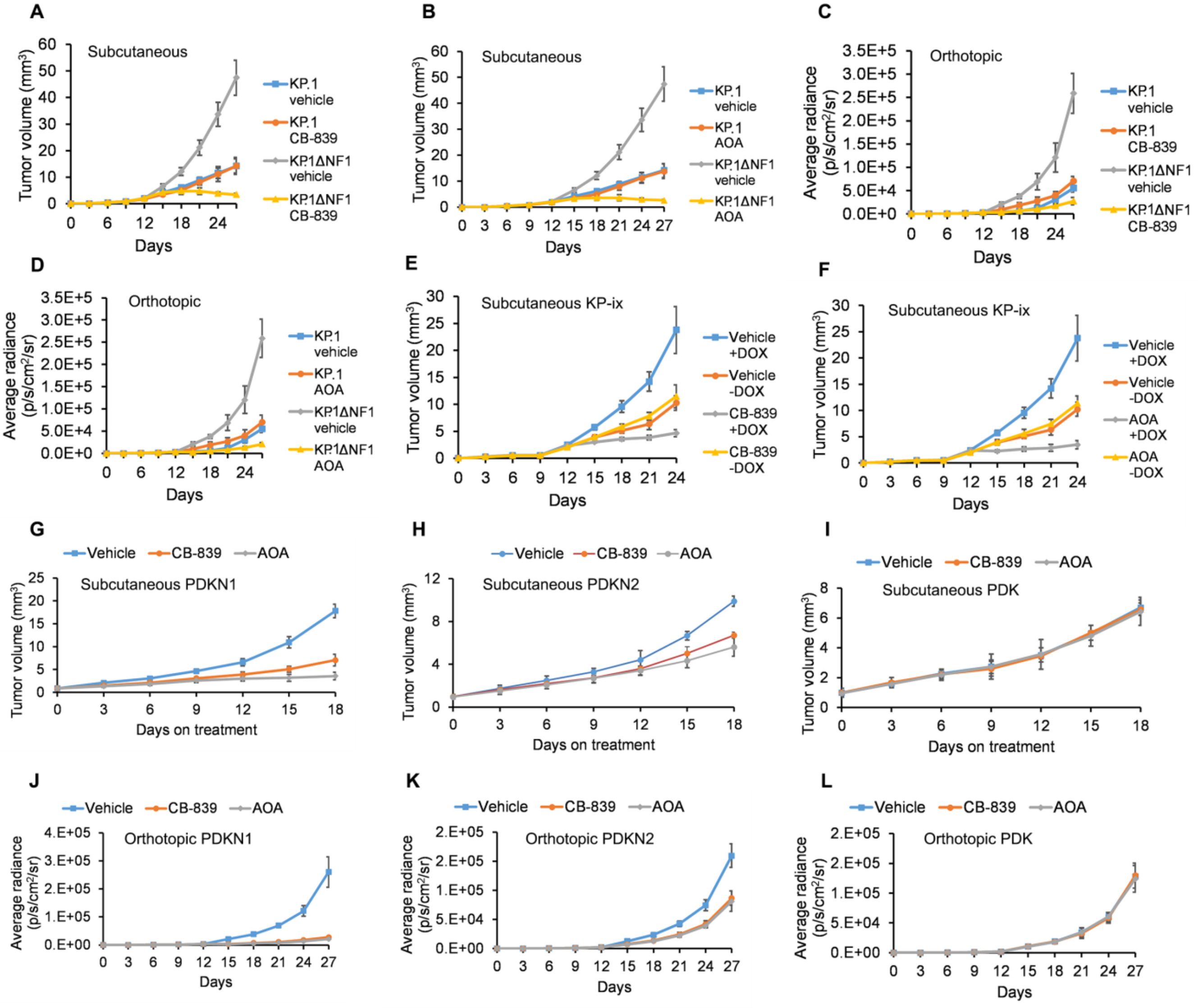

Supplement: Supplementary file 1 — Appendix [file EMMM-11-e9856-s001.pdf]
