# Supplementary figures and images for: Nf1 loss promotes Kras‐driven lung adenocarcinoma and results in Psat1‐mediated glutamate dependence
Source: EMBO Mol Med. 2019 Apr 29;11(6):e9856. doi: 10.15252/emmm.201809856 (PMC6554671; doi:10.15252/emmm.201809856)

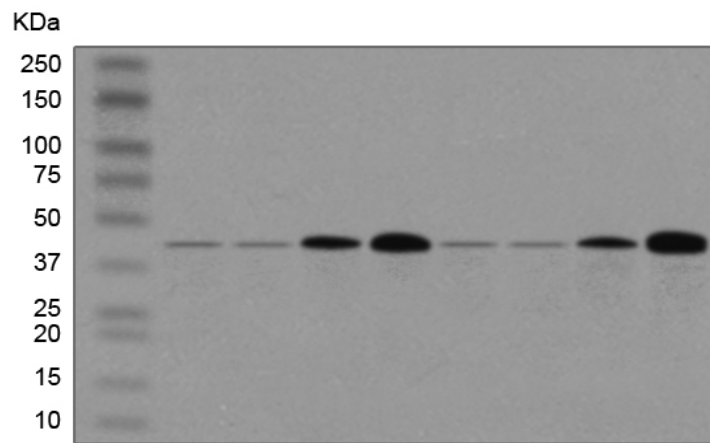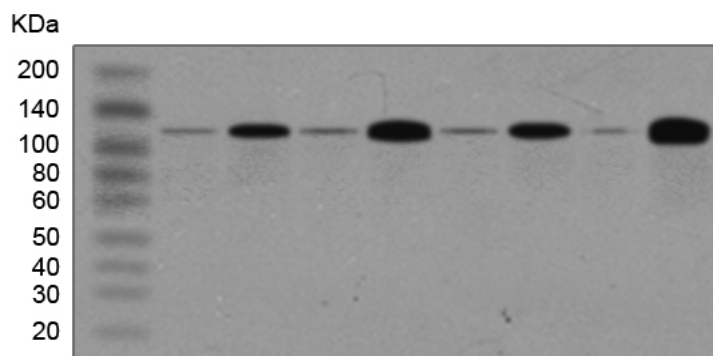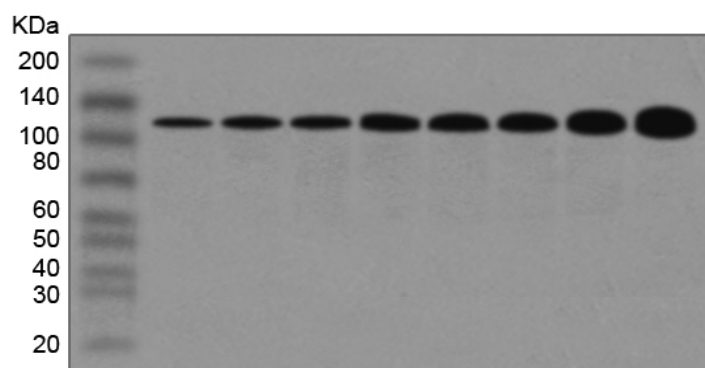

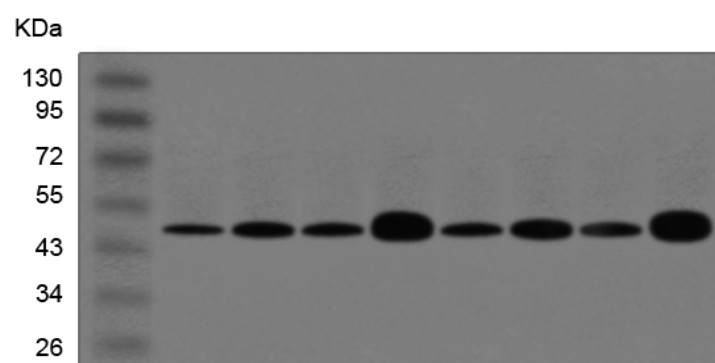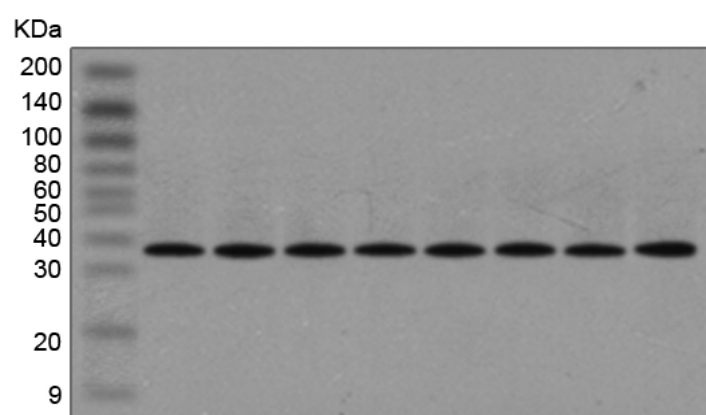

Supplement: Supplementary file 3 — Source Data for Appendix [file EMMM-11-e9856-s005.zip › AppendixFigs/S6a.pdf]

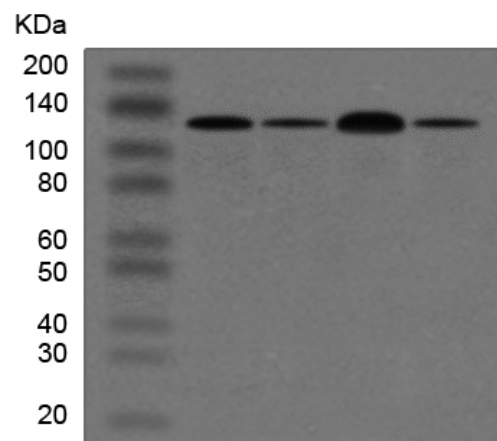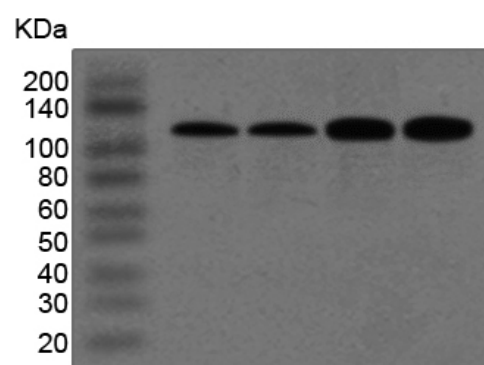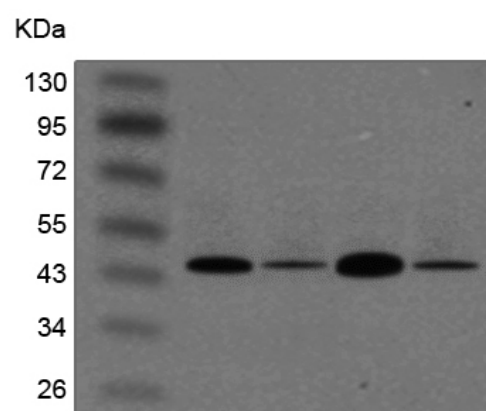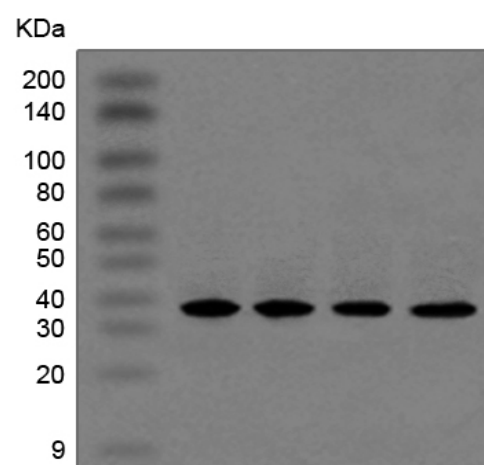

Supplement: Supplementary file 3 — Source Data for Appendix [file EMMM-11-e9856-s005.zip › AppendixFigs/S6g.pdf]

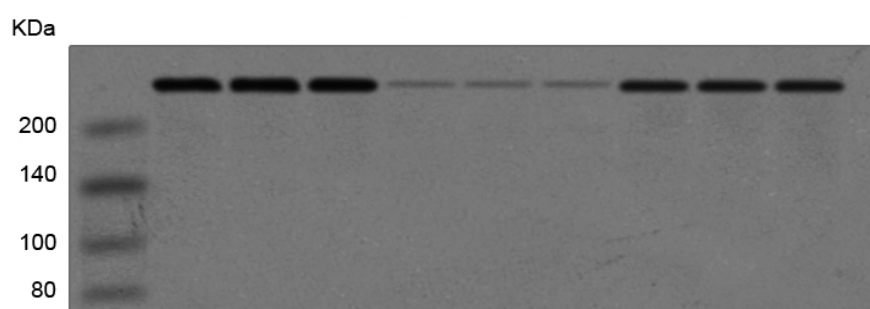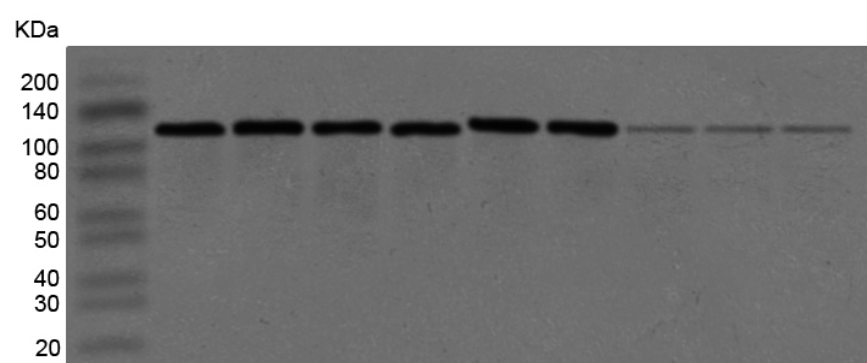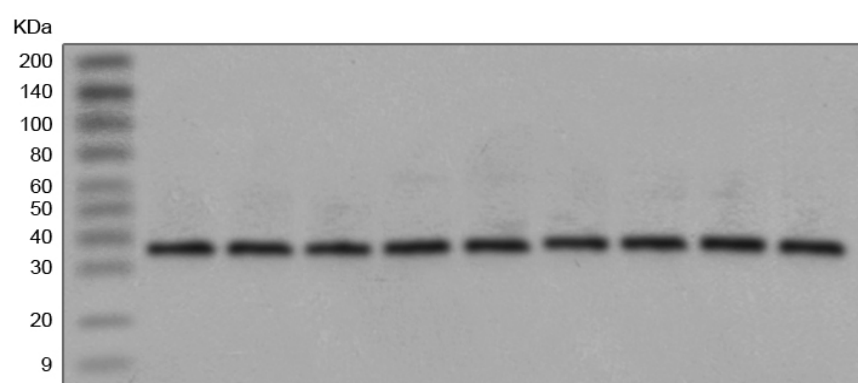

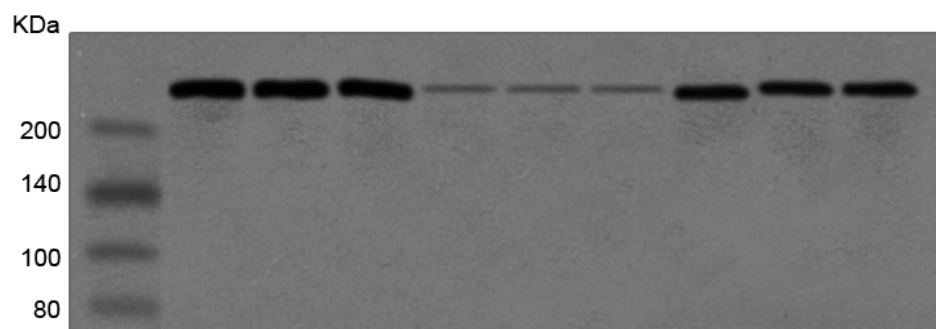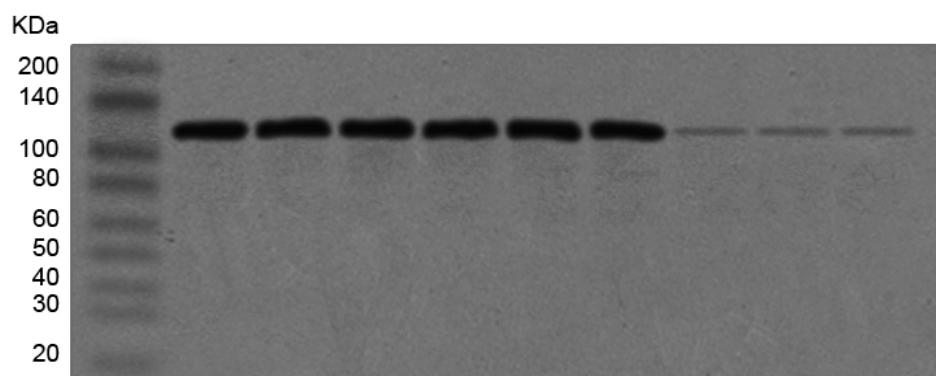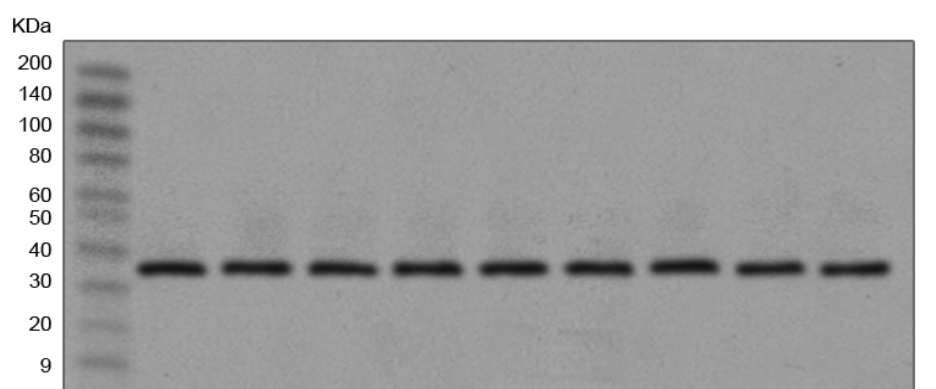

Supplement: Supplementary file 3 — Source Data for Appendix [file EMMM-11-e9856-s005.zip › AppendixFigs/S1b.pdf]

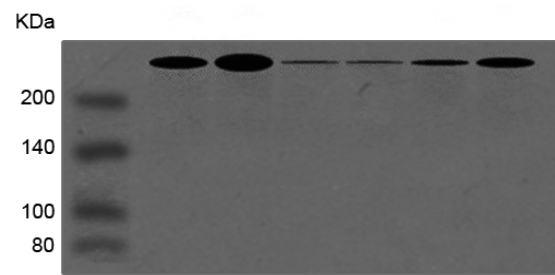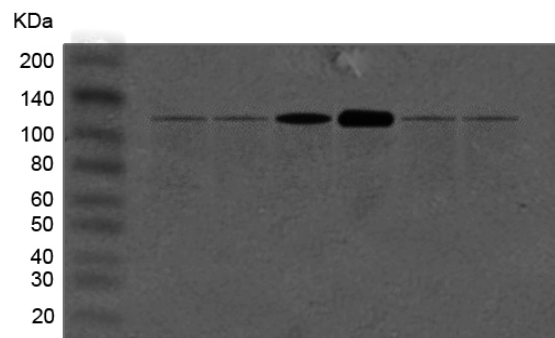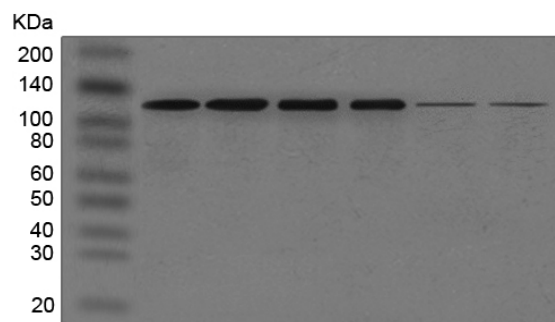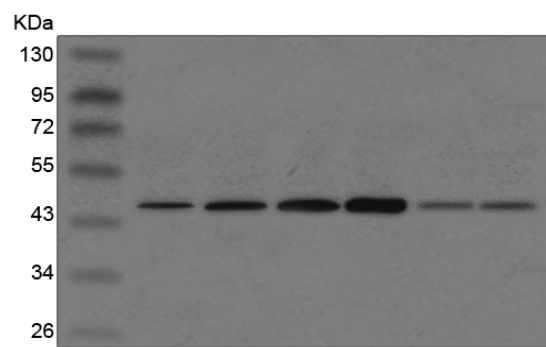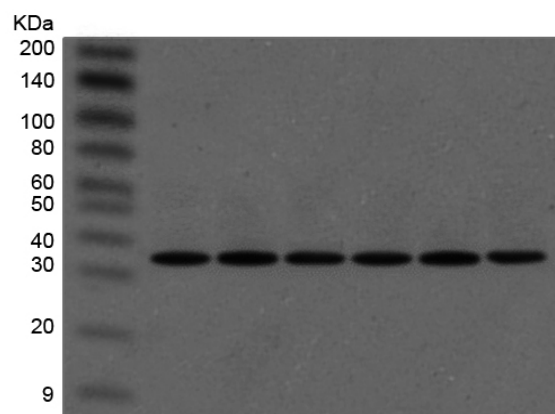

26.03.19

Supplement: Supplementary file 3 — Source Data for Appendix [file EMMM-11-e9856-s005.zip › AppendixFigs/S3a.pdf]

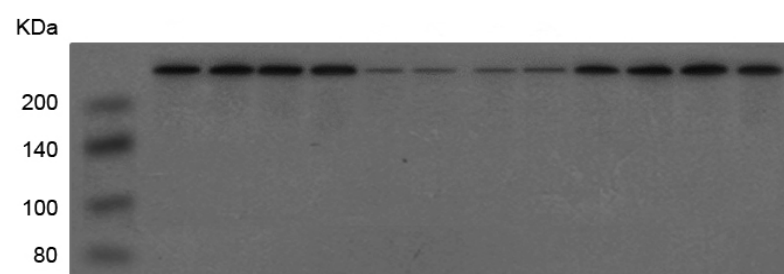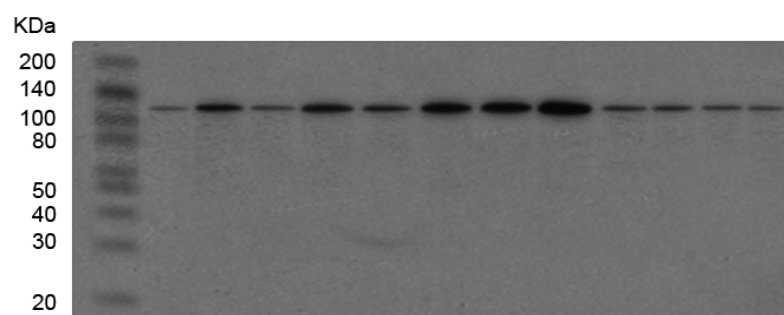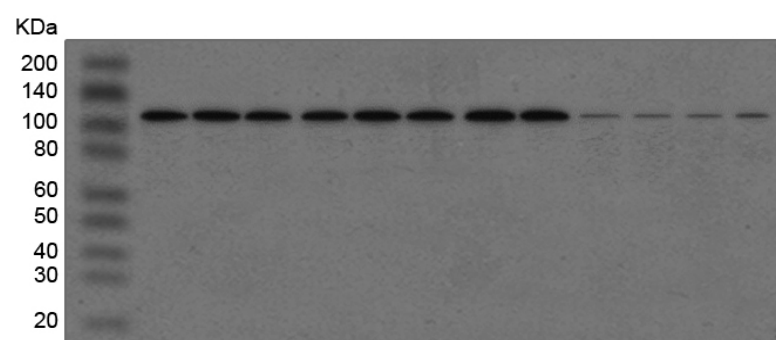

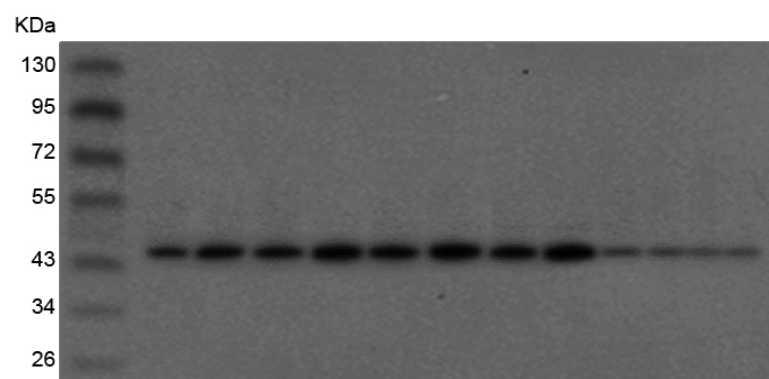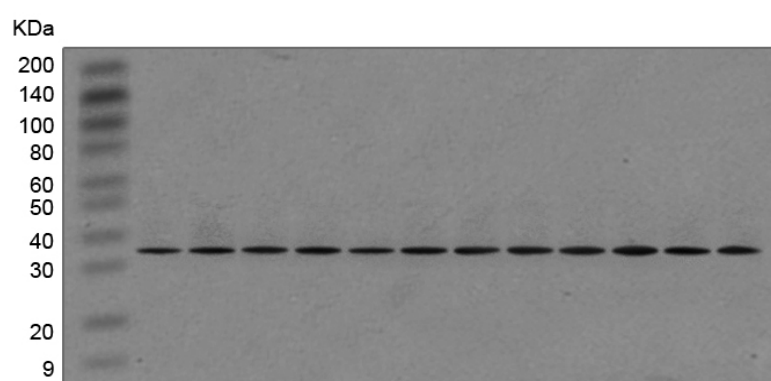

Supplement: Supplementary file 3 — Source Data for Appendix [file EMMM-11-e9856-s005.zip › AppendixFigs/S3c.pdf]

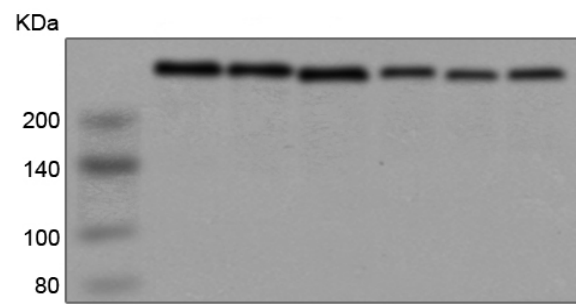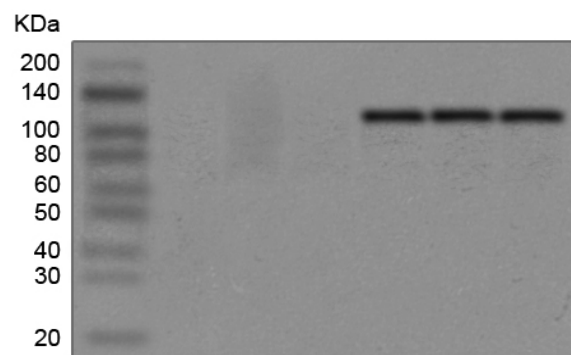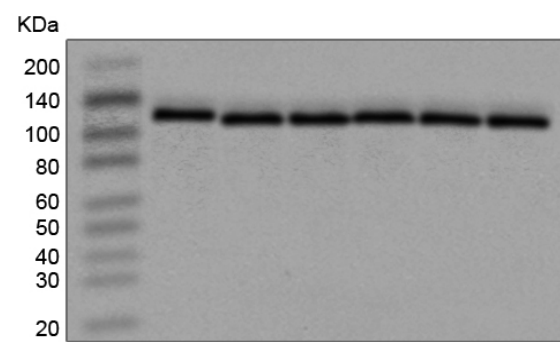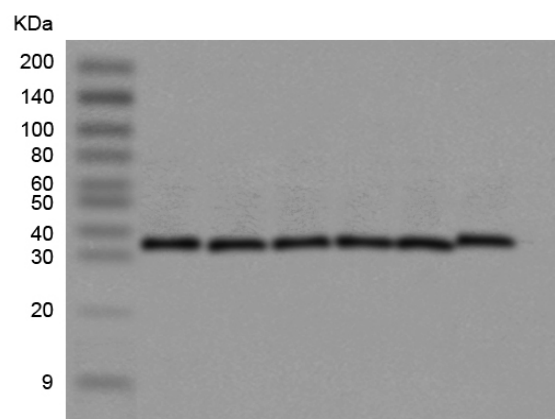

Supplement: Supplementary file 5 — Source Data for Figure 2 [file EMMM-11-e9856-s003.zip › Fig2/2e.pdf]

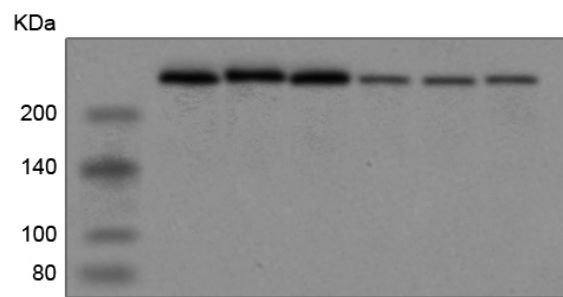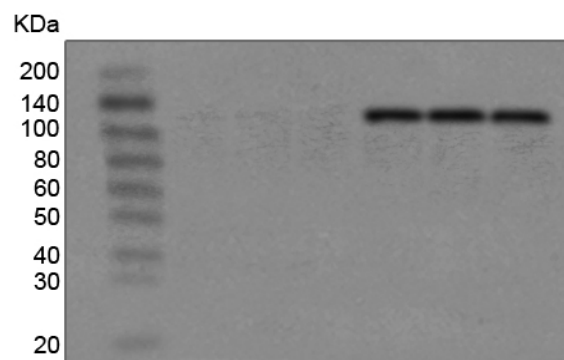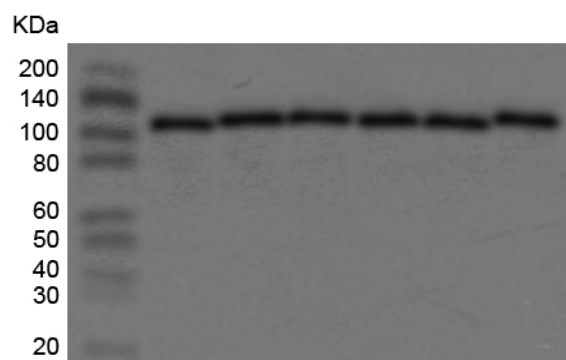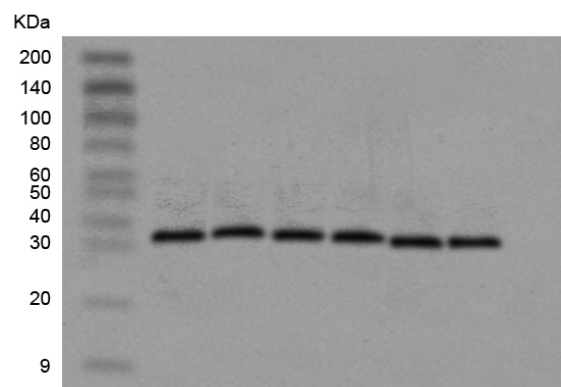

Supplement: Supplementary file 5 — Source Data for Figure 2 [file EMMM-11-e9856-s003.zip › Fig2/2d.pdf]

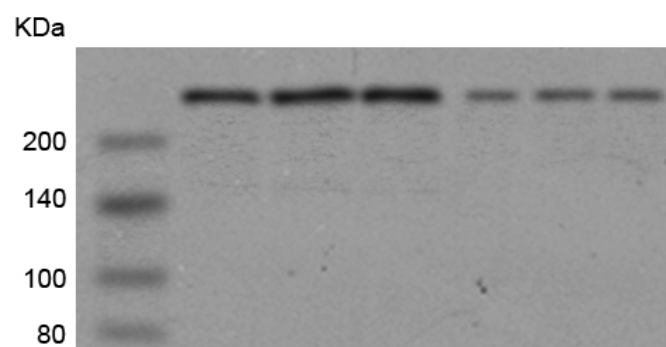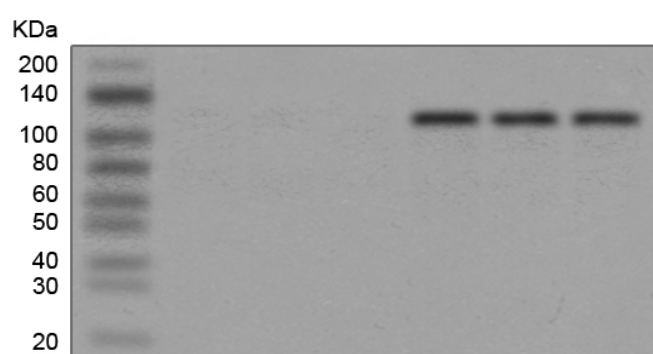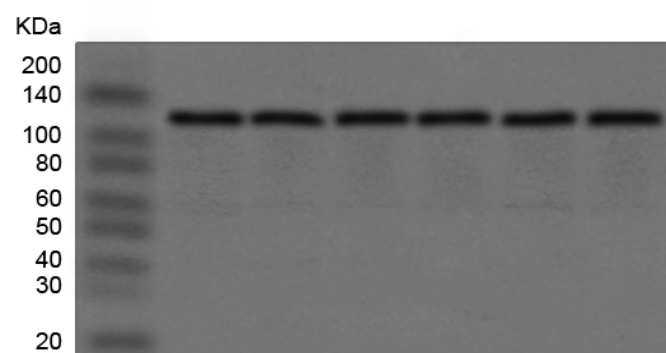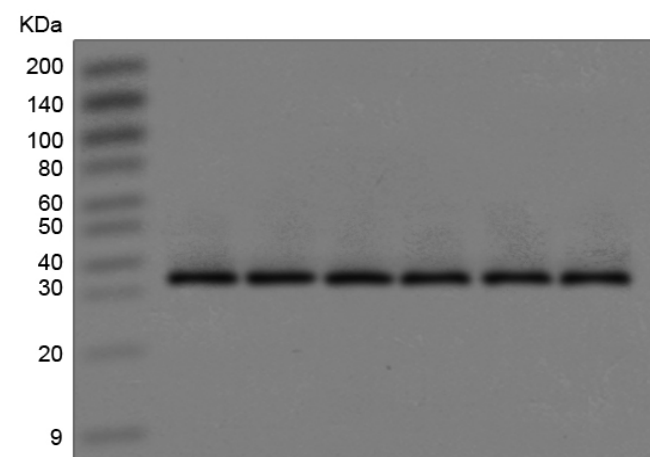

Supplement: Supplementary file 5 — Source Data for Figure 2 [file EMMM-11-e9856-s003.zip › Fig2/2f.pdf]

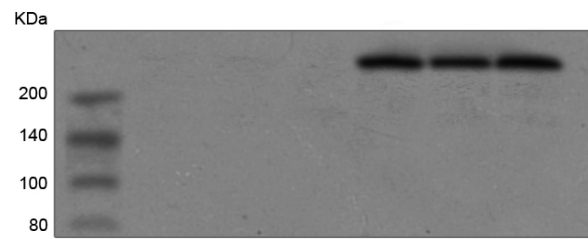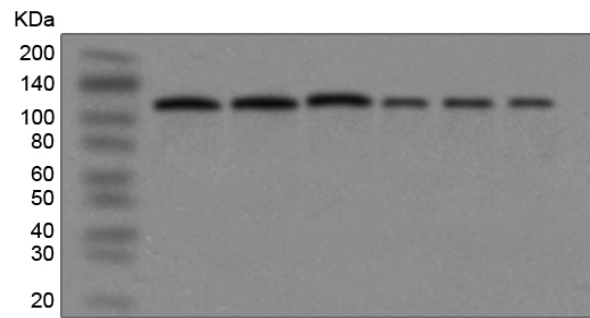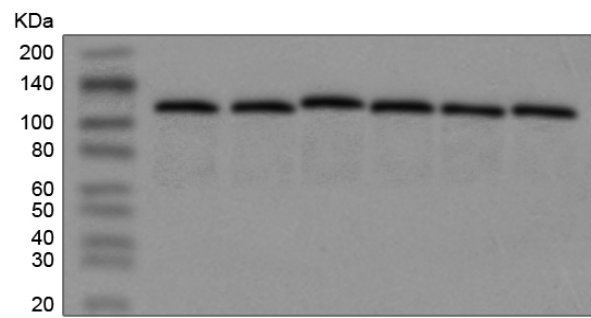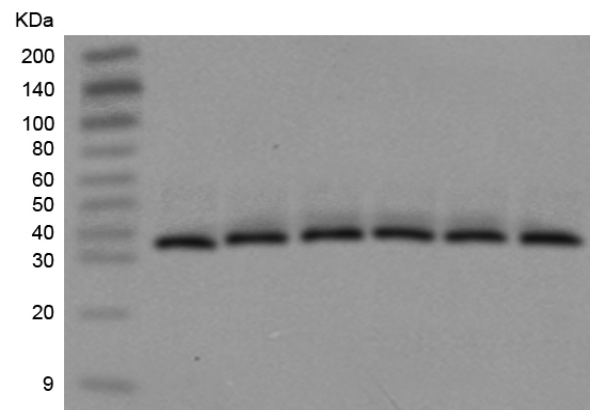

Supplement: Supplementary file 5 — Source Data for Figure 2 [file EMMM-11-e9856-s003.zip › Fig2/2c.pdf]

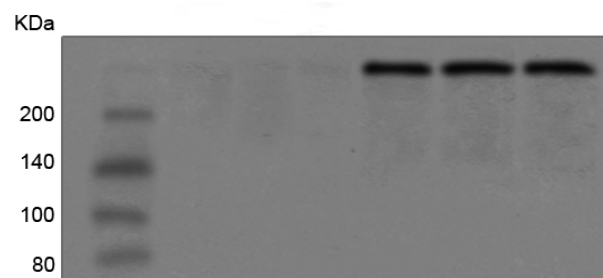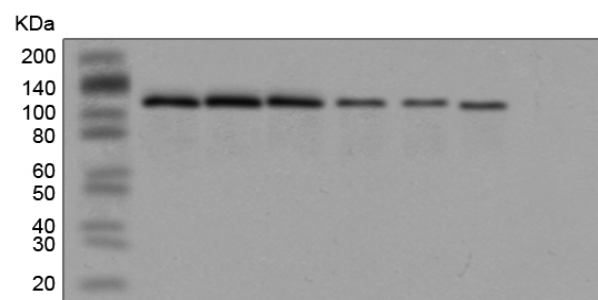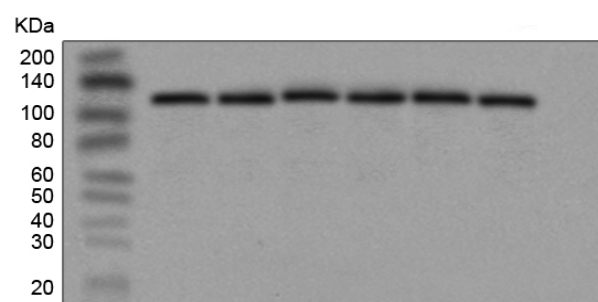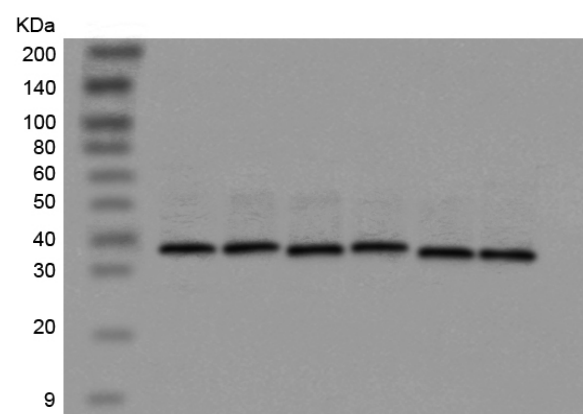

Supplement: Supplementary file 5 — Source Data for Figure 2 [file EMMM-11-e9856-s003.zip › Fig2/2b.pdf]

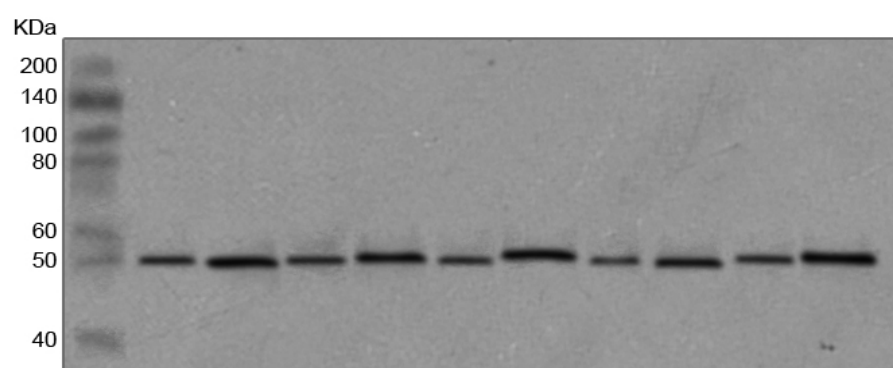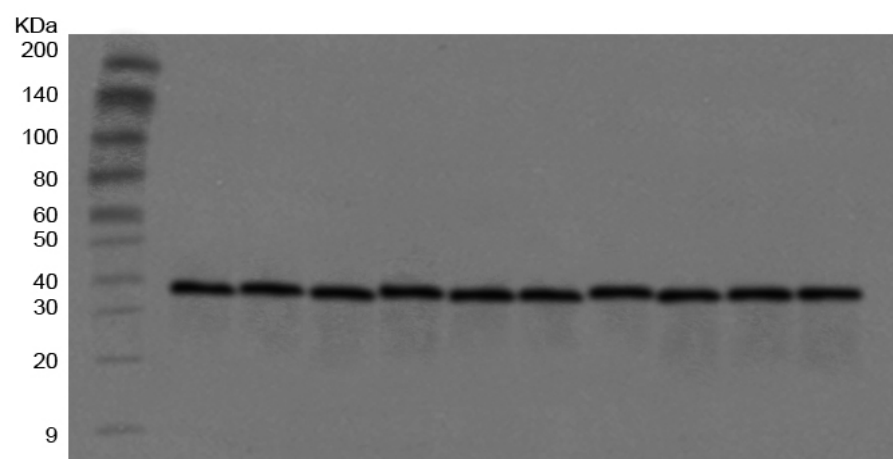

Supplement: Supplementary file 5 — Source Data for Figure 2 [file EMMM-11-e9856-s003.zip › Fig2/2a.pdf]

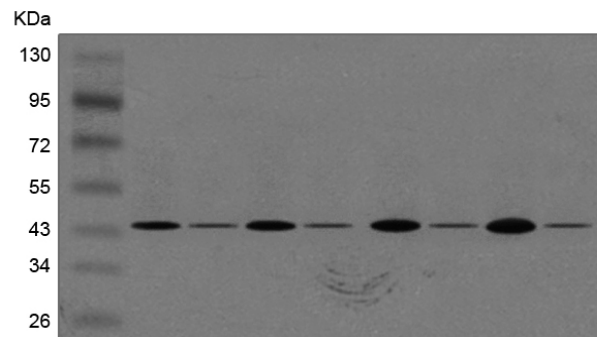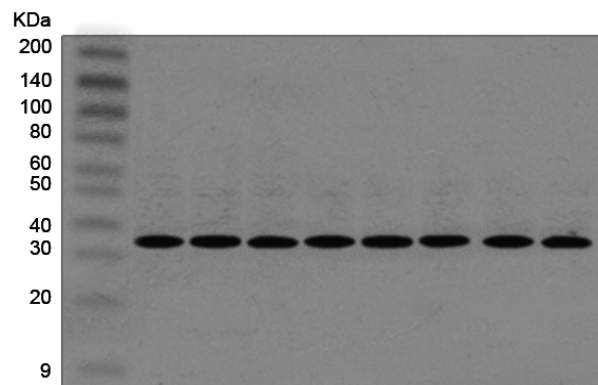

Supplement: Supplementary file 6 — Source Data for Figure 3H [file EMMM-11-e9856-s004.pdf]
